# Regensburg Insomnia Scale (RIS): a new short rating scale for the assessment of psychological symptoms and sleep in insomnia; Study design: development and validation of a new short self-rating scale in a sample of 218 patients suffering from insomnia and 94 healthy controls

**DOI:** 10.1186/1477-7525-11-65

**Published:** 2013-04-22

**Authors:** Tatjana Crönlein, Berthold Langguth, Roland Popp, Helmut Lukesch, Christoph Pieh, Göran Hajak, Peter Geisler

**Affiliations:** 1 Department of Psychiatry and Psychotherapy, University Regensburg, Universitaetsstrasse 84, Regensburg, 93042, Germany; 2 Department for Psychology, University Regensburg, Universitaetsstrasse 31, Regensburg, 93053, Germany

**Keywords:** Sleep, Psychophysiological insomnia, Psychological symptoms, Scale, PSQI

## Abstract

**Background:**

The Regensburg Insomnia Scale (RIS) is a new self-rating scale to assess cognitive, emotional and behavioural aspects of psychophysiological insomnia (PI) with only ten items. A specific purpose of the new scale is the evaluation of the outcome of insomnia- specific cognitive behaviour therapy (CBT-I).

**Methods:**

Internal consistency of the RIS has been validated in 218 patients with PI. For determining sensitivity and specificity, this sample has been compared to 94 healthy controls. Sensitivity to change and pre-post cross-validation with the Pittsburgh Sleep Quality Index (PSQI) has been tested in a separate sample of 38 patients with PI undergoing CBT-I.

**Results:**

RIS distinguishes well between controls and patients with PI. Internal consistency was within a good range (Cronbach alpha = .890). RIS was sensitive for detecting improvements after CBT-I in sleep parameters and target symptoms such as sleep-related thinking.

**Conclusion:**

The RIS is a valid and feasible instrument for assessing psychological PI-symptoms and sleep parameters.

## Background

Insomnia is a frequent disorder with prevalences varying between 2% [1] and 48% [2] depending on the diagnostic criteria used. It is often associated with psychiatric and organic diseases and produces costs due to accidents and absentieesm [3-5]. Several tools for measuring insomnia have been developed [6-9] reflecting the growing interest and need for assessment instruments in insomnia research. However, there still is a need for a short scale measuring the psychological aspects of insomnia together with quantitative aspects of sleep.

Why measure the psychological aspects of insomnia? The reason is that there is growing evidence that psychological symptoms are prominent, perpetuating factors for disturbed sleep [10,11], which can be best seen in psychophysiological insomnia [12] (PI). Psychological aspects such as sleep related worries and rumination, dysfunctional attitudes toward sleep [13], distorted sleep perception [14] and hyperarousal [15] have been found and investigated thoroughly. It is assumed that insomnia-specific hyperarousal [16] is fuelled by dysfunctional beliefs (such as “I have to sleep at least 8 hours per night”) and by the anticipating the negative consequences of sleep loss. Thus, while trying to fall asleep, enhanced self observation occurs, which in return has an impairing effect on sleep onset [17]. The relevance of psychological aspects in the continuance of chronic insomnia is increasingly being recognized and specific psychotherapeutic treatments such as Cognitive Behaviour Therapy for Insomnia (CBT-I) have been developed and tested [18-20]. The psychological impact makes it plausible, that PI is comorbid in other sleep disorders such as sleep apnea [21] or Periodic Leg Movement in sleep [22] and the co-existence explains why sleep will not improve by treating the organic disorder [21] alone. In order to assess the psychophysiological components in sleep disorders, a short scale would be useful in clinical routine.

Indeed, there are already a variety of scales assessing psychological aspects of insomnia. For measuring the state of cognitive and somatic arousal there is the Pre-Sleep Arousal Scale [23], the Presleep Cognitive Activity Questionnaire [24] and the Arousal Level as Present State [25]. The Metacognitions Questionnaire-Insomnia (MSQ-I) [26] and the Dysfunctional and Attitudes about Sleep scale [27] have been developed to measure insomnia-specific worrisome thoughts and beliefs. The Thought Control Questionnaire-Insomnia revised scale [28] measures the attempts to control unwanted thoughts in insomnia. The Utility of Presleep Worry Questionnaire (UPWQ) was developed to investigate the utility of presleep worry in insomnia [29] as well as the Sleep Disturbance Questionnaire [30]. The Monitoring for Sleep-Related Threat [31] measures the stimuli that might hinder falling asleep. The Glasgow Sleep Effort Scale was designed to measure the attempt to control the process of falling asleep [32]. All these scales cover psychological aspects of insomnia, however they are too specific for clinical routine, since they each just measure certain symptoms, such as arousal state or presleep worrying.

The Insomnia Severity Index (ISI) [6] is the most established instrument for measuring insomnia symptoms, however there is only one out of 7 items that targets the psychological symptoms, namely being worried about the current sleep pattern. Violani’s Insomnia Scale [9] covers different items such as medication intake, snoring, consulting a doctor, in addition to sleep related items, however this scale does not address clinical psychophysiological symptoms of insomnia and was rather designed for the pre-screening of subjects with insomnia complaints.

A short scale covering quantitative aspects of sleep as well as insomnia specific symptoms is still lacking. Thus we aimed at developing a short new instrument for the assessment of the typical psychological aspects of PI that can be especially used for the evaluation of therapeutic interventions. This scale was validated using a large sample of insomnia patients and healthy controls. Moreover we evaluated the scale’s sensitivity to therapeutic changes in patients with PI who were treated with CBT-I in our hospital. The Regensburg Insomnia Scale (RIS) was administered before and 6 months after completion of therapy. The study was approved by the Ethics Committee of the University of Regensburg.

## Methods

### Construction of the scale

The RIS was designed in German. In a pre-study, three sleep experts who routinely work with insomnia patients compiled typical complaints of PI patients. Emphasis was placed on the exact wording, such as “I wake up from the slightest sound”. To identify insomnia specific items, this list was given to patients with sleep-apnea (N = 33), insomnia (N = 36) and to healthy controls (N = 29). Items that did not discriminate between insomnia patients and controls or between insomnia and sleep apnea patients (Mann-Whitney-Test) were eliminated. The remaining list was further shortened to the demands of a short and practicable scale measuring sleep and cognitive, emotional and behavioural symptoms of PI. Five items were selected to cover quantitative and qualitative sleep parameters: Sleep latency (1), sleep duration (2), sleep continuity (3), early awakening (4) and sleep depth (5). Four items ask about the psychological aspects of PI, such as the experience of sleepless nights (6), focussing on sleep (7), fear of insomnia (8), and daytime fitness (9), one item is about sleep medication (10). A 5-step Likert scale was provided for response. This type of scale was also used for quantitative sleep parameters (sleep duration and sleep latency), because, according to our clinical experience, insomnia patients have difficulties giving exact answers when asked about quantitative data.

The total score ranges from 0 to 40 points. The introductory question regarding bedtime hours is not included in the score. It serves as a plausibility check (for example to check whether time in bed matches duration of sleep and sleep latency). After validation of the German version, the English version of the RIS was created by sleep experts fluent in both languages using back-and-forth translation (Table 1).

**Table 1:** Regensburg insomnia scale

**PLEASE RATE THE FOLLOWING QUESTIONS FOR THE LAST FOUR WEEKS**					
***DIE FRAGEN BEZIEHEN SICH AUF DIE LETZTEN VIER WOCHEN***					
**0 AT WHAT TIME DO YOU USUALLY GO TO BED?**	**WHEN DO YOU USUALLY GET UP?**				
**0.*MEINE ÜBLICHEN BETTZEITEN SIND VON***	***UHR BIS***		***UHR***		
1. How many minutes do you need to fall asleep?	1–20 min.	21–40 min.	41–60 min.	61–90 min.	91 min. and more
*1. Wie viele Minuten brauchen Sie zum Einschlafen?*	0	1	2	3	4
2. How many hours do you sleep during the night?	7 h and more	5–6 h	4 h	2–3 h	0–1 h
*2. Wie viele Stunden schlafen Sie in der Nacht?*	0	1	2	3	4
How often do the following occurrences happen?	Always	Mostly	Sometimes	Seldom	Never
*Wie oft treffen folgende Ereignisse zu?*	*Immer*	*Meistens*	*Manchma*l	*Selten*	*Nie*
3. My sleep is disturbed	4	3	2	1	0
*3. Ich kann nicht durchschlafen*					
4. I wake up too early	4	3	2	1	0
*4. Ich wache zu früh auf*					
5. I wake up from the slightest sound	4	3	2	1	0
*5. Ich wache schon bei leichten Geräuschen auf*					
6. I feel that I have not slept all night	4	3	2	1	0
*6. Ich habe das Gefühl, die ganze Nacht kein Auge zugetan zu haben*					
7. I think a lot about my sleep	4	3	2	1	0
*7. Ich denke viel über meinen Schlaf nach*					
8. I am afraid to go to bed because of my disturbed sleep	4	3	2	1	0
*8. Ich habe Angst ins Bett zu gehen, da ich befürchte nicht schlafen zu können.*					
9. I feel fit during the day	0	1	2	3	4
*9. Ich fühle mich voll leistungsfähig*					
10. I take sleeping pills in order to get to sleep	4	3	2	1	0
*10. Ich nehme Schlafmittel, um einschlafen zu können*					

English and German version.

### Sample

After construction of the scale test properties have been investigated in two separate samples of patients with PI: one sample in which normative data (NORM) were gathered and one additional sample of patients who were tested before and after CBT-I (THERAPY). Patients were recruited from inpatients and outpatients of the Center for Sleep Medicine of the Department of Psychiatry and Psychotherapy, University Regensburg (Germany). All patients have been seen by a psychiatrist and a psychotherapist. The inclusion criterion for the insomnia patient sample was a diagnosis of PI according to International Classification of Sleep Disorders-2 (ICSD-2) [12], irrespective of the intake of hypnotics. In case a sleep apnea or Periodic Limb Movements in Sleep was suspected a monitoring with an apnea screening instrument or a high resolution actigraphy respectively would be done. Inpatients had a routine polysomnography. Current or past continuous shift work or night work and current severe physical or mental disorders with a major influence on sleep were exclusion criteria. All participants signed informed consent forms.

NORM was a sample of 218 PI patients with a mean age of 48.9 ± 13.8 yrs (88 males, mean age 50.0 ± 13.4 yrs; 130 females, mean age: 48.0 ± 14.0 yrs) and a prior mean duration of insomnia of 9.5 ± 9.5 yrs. 33 patients were inpatients admitted for treatment of severe chronic insomnia, 47 inpatients were admitted for diagnostic purposes and 138 patients were recruited from the outpatient clinic. All patients were seeking help in specialized center for sleep.

THERAPY consisted of 30 women (mean age: 57.7 ± 11.3 yrs) and 8 men (mean age 51.1 ± 11.2 yrs) who participated in a standardized CBT-I program in our sleep center [33,34] as inpatients. 21 patients were taking hypnotics prior to admission. Patients agreed to discontinue all sleep medication during the program. The CBT-I program is a standardized two-week program based on current psychotherapy standards for insomnia and includes polysomnography, bedtime restriction, relaxation therapy, stimulus control therapy and a psychoeducational component aimed at correcting dysfunctional beliefs [10,13]. All patients completed a RIS and a Pittsburgh Sleep Quality Index (PSQI) [35] at baseline and 6 months later.

To assess specificity and sensitivity, a sample of 94 healthy controls were investigated (mean age: 46.8 ± 12.9 yrs; 42 men; mean age: 50.0 yrs ± 13.0; 52 women; mean age 44.2 ± 12.3 yrs). The control sample was recruited from relatives of patients, the hospital staff and their relatives in different cities of Germany. Persons in all test samples were different from those patients and controls that were tested in the construction period. Controls also filled out a PSQI. Exclusion criteria were: 1. current or past complaint of disturbed sleep or excessive daytime sleepiness 2. current or past continuous shift work or night work; 3. intake of hypnotics, 4. current severe physical or mental disorder with a major influence on sleep. No differences in age and sex distribution were found between PI patients (NORM) and controls (students’ *t*-test, n.s.).

### Test properties

Normative data (mean scores and mean item scores) were gathered from the NORM group and controls. Cronbachs alpha as well as a corrected item-total correlation were calculated. Specificity and sensitivity was tested in the samples of PI and controls for RIS and for PSQI separately. A component analysis was done with Varimax rotation in the insomnia sample. The loading cut-off chosen to determine which items loaded on a factor was 0.6.

In order to measure sensitivity of the RIS for therapy-related changes, the RIS total scores, single items and PSQI scores (total and subscales) before and after therapy were compared with participants’ *t*-test. The effect sizes were calculated with Cohen’s d.

## Results

### Discriminative power

**Figure 1:**
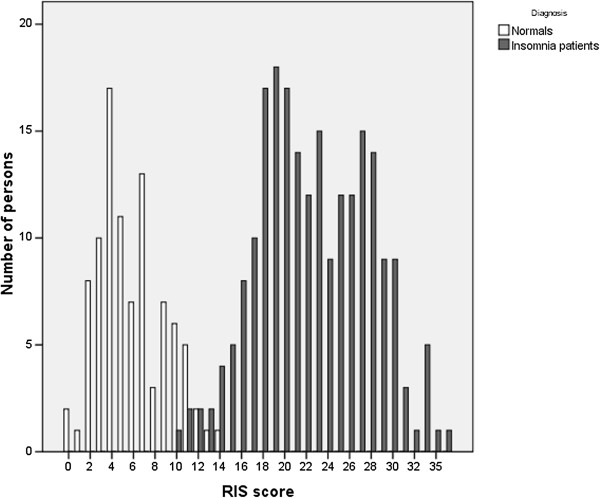
Distribution of RIS scores of 218 patients with psychophysiological insomnia and 94 controls.

The mean RIS score for the sample was 22.6 points with a standard deviation of 5.19. The mean score for single items ranged from 1.65 (item 2 “short sleep duration”) to 3.11 (item 3 “disturbed sleep continuity”). The majority of responses for all items were in the range from 2 to 4 points, which points to relevant pathology. The full range of possible responses was used for all items (Table 2). The RIS score distribution showed a clear bimodal distribution with a relatively small overlap between 10 and 14 points (Figure 1). The median score of 22 in PI patients is significantly higher than the score of the control group (5 points, Mann-Whitney U = 36; p < .0005). With a cut-off score of 12 points, sensitivity was 97.7% for insomnia patients and specificity was 97.9% for the normal control sample. Sensitivity of PSQI (cut-off score = 6 points) was 98% and specificity was 98%.

**Table 2:** Means and standard deviations and percentages in RIS in 218 patients with psychophysiological insomnia

**RIS Items**	**Mean (SD)**	**Answer distribution (percentage)**					**Corrected item-total correlation**
		1–20 min.	21–40 min.	41–60 min.	61–90 min.	91 min. and more	
**1. Sleep latency**	1.82 (1.37)	*21.1*	*24.8*	*21.6*	*16.1*	*16.5*	.549
		7 h and more	5–6 h	4 h	2–3 h	0–2 h	
**2. Sleep duration**	1.65 *(0.86)*	*7.3*	*37.2*	*40.8*	*12.8*	*1.8*	.694
		**Never**	**Seldom**	**Sometimes**	**Mostly**	**Always**	
**3. My sleep is disturbed**	3.11 *(0.97)*	*0.5*	*7.3*	*17.4*	*30.7*	*44.0*	.732
**4. I wake up too early**	2.86 *(1.03)*	*2.8*	*7.3*	*22.9*	*35.3*	*31.7*	.650
**5. I wake up from the slightest sound**	2.77 *(1.08)*	*3.2*	*10.1*	*23.4*	*33.5*	*29.8*	.619
**6. I feel that I have not slept all night**	2.07 *(0.97)*	*6.9*	*17.0*	*44.0*	*26.1*	*6.0*	.748
**7. I think a lot about my sleep**	2.41 *(0.92)*	*2.8*	*11.5*	*37.6*	*38.1*	*10.1*	.673
**8. I am afraid to go to bed because of my disturbed sleep**	1.89 *(1.24)*	*17.0*	*21.1*	*29.8*	*20.6*	*11.5*	.676
**9. I feel fit during the day**	2.31 (1.03)	*2.3*	*23.9*	*26.6*	*35.3*	*11.9*	.508
**10. I take sleeping pills in order to get to sleep**	1.7 (1.56)	*35.8*	*12.4*	*18.8*	*12.4*	*20.6*	.519

* Item-total correlations relate to the full scale and not the four subscales.

### Reliability

Cronbach’s alpha was calculated for the whole sample. With an alpha of .890 internal consistency was good. Corrected item-total correlation with the full scale showed high correlation coefficients for all items (see Table 2).

### Dimensions of the scale

Component analysis with Varimax rotation was performed in the NORM sample. Statistical analyses were performed with SPSS 15 (SPSS Inc.). Rotated component analysis revealed four components (Table 3). The first component comprises the items 3–5 (“disturbed sleep continuity”, “easy awakening” and “early awakening”) and is labelled “sleep depth”. It explains 20.56% of the variance (Eigenvalue: 2.44). The second component explains 18.52% of the variance (Eigenvalue: 1.78); it includes items 1, 2 and 6, which are related to sleep quantity (“prolonged sleep latency”, “short sleep duration” and “sleepless nights”) and is called “sleep quantity”. The third component includes items 7 and 8 (“thinking about sleep” and “fear of insomnia”) and explains 14.95% of the variance (Eigenvalue: 1.31), it is labelled “fearfully focussing on insomnia”. The last factor includes the items 9 and 10 (“impaired daytime fitness” and “hypnotics intake”) and explains 12.0% of the variance (Eigenvalue: 1.07). It is called “hypnotics and daytime functioning”.

**Table 3:** Component analysis with Varimax rotation of the RIS in 218 insomnia patients

**RIS Items**	**I**	**II**	**III**	**IV**	**Communalities**
1. Sleep latency	−.29	.70	.01	.33	0.68
2. Sleep duration	.44	.69	−.10	−.08	0.69
3. Sleep continuity	.81	.08	−.01	.04	0.66
4. Early awakening	.79	−.04	.00	.02	0.63
5. Easy awakening	.65	.10	−.14	−.08	0.47
6. Sleepless nights	.19	.71	.26	−.27	0.68
7. Thinking about sleep	.15	−.14	.90	.02	0.85
8. Fear of insomnia	−.07	.42	.74	.13	0.75
9. Impaired daytime	.03	.25	.04	−.73	0.61
10. Hypnotic intake	.05	.29	.19	.67	0.58
Variance	20.56%	18.52%	14.95%	12.01%	

Component I: Sleep depth, Component II: Sleep quantity, Component III: Fearfully focussing on insomnia and component IV: Sleep medication/daytime functioning.

### Treatment effects

Both RIS and PSQI were sensitive for the effects of CBT-I in the THERAPY sample (see Table 4). All items of RIS except “sleep duration” (item 2) were significantly improved. In the PSQI there was a significant reduction in all subscale scores except for duration of sleep, sleep disorders and daytime fitness. Cohen’s d revealed strong effects for RIS item 10”hypnotics intake” (d = 0.95) after six months, this effect is also seen in the PSQI (Cohen’s d = 0.97). Effects on sleep latency are comparable in RIS and PSQI with Cohen’s d = 0.44 and 0.45 respectively. Both scales are also comparable regarding effects on sleep duration. The PSQI reveals a medium effect on sleep parameters in subscales sleep quality and sleep efficiency. The effects on sleep parameters in RIS are negligible. The RIS reveals a major effect in item 7 “thinking about sleep” and minor effects in “fear of insomnia” (item 8) and “sleepless nights” (item 6).

**Table 4:** Pre post data of 38 patients with PI who had been treated with CBT-I

**Item**	**Baseline**	**Post (6 months)**	**Participants’*t*-test**	**Effect size d**
	Mean (SD)	Mean (SD)		
*RIS score*	*23.45 (4.8)*	*16.05 (5.36)*	*9.52 (p < .0005)*	*0.37*
1. Sleep latency	1.87 (1.39)	1.13 (0.87)	3.37 (p = .002)	0.44
2. Sleep duration	1.87 (1.12)	1.68 (1.22)	n.s.	
3. Sleep continuity	3.37 (1.12)	2.97 (1.22)	2.57 (p = .014)	0.29
4. Early awakening	3.16 (1.03)	2.79 (1.02)	2.67 (p = .011)	0.36
5. Easy awakening	2.55 (1.15)	2.11 (1.20)	2.67 (p = .011)	0.37
6. Sleepless nights	1.82 (1.04)	1.39 (0.95)	2.20 (p = .034)	0.43
7. Thinking about sleep	2.24 (0.92)	1.68 (0.93)	3.70 (p = .001)	0.78
8. Fear of insomnia	1.61 (1.17)	1.18 (1.11)	2.46 (p = .019)	0.32
9. Impaired daytime	2.37 (1.02)	2.00 (1.15)	2.06 (p = .046)	0.32
10. Hypnotic intake	2.61 (2.60)	0.79 (0.79)	6.12 (p < .0005)	0.95
*PSQI score*	*14.37 (2.52)*	*11.29 (3.52)*	*5.56 (p < .0005)*	*0.34*
1. Sleep quality	2.05 (0.65)	1.66 (0.81)	3.58 (p = .001)	0.60
2. Sleep latency	2.16 (1.05)	1.66 (1.05)	3.24 (p = .003)	0.45
3. Sleep duration	2.68 (0.57)	2.47 (0.73)	n.s.	
4. Sleep efficiency	2.63 (0.63)	2.18 (0.98)	2.82 (p = .008)	0.61
5. Sleep disorders	1.21 (0.53)	1.34 (0.53)	n.s.	
6. Hypnotics intake	2.08 (1.30)	0.66 (1.10)	6.50 (p < .0005)	0.97
7. Daytime sleepiness	1.55 (0.92)	1.31 (0.84)	n.s.	n.s.

Means and standard deviation of scores and items of RIS and subscales of PSQI. Cohen’s d values for effect sizes of CBT-I.

### Feasibility

No participant reported problems in understanding or completing the scale. Completion of the scale took the patients an average of approximately two minutes. The score can be calculated within less than a minute. Thus both completion and evaluation of the scale is much less time consuming as compared to other scales, e.g. the Pittsburgh Sleep Quality Index (PSQI).

## Discussion

The RIS is a new self-rating scale with ten items, developed to measure symptoms of PI with special emphasis on psychological symptoms.

The scale covers the characteristic cognitive, emotional and behavioural aspects of PI. These items are essential target symptoms in CBT-I [36,37]. With an average time for completion and evaluation of less than three minutes, it is a highly efficient tool both for research and clinical practice. The RIS discriminates well between PI and healthy controls. We propose to consider a score from 0–12 points as normal and a higher score as indicative of PI symptoms.

The relatively high sensitivity and specificity values of our scale may be explained by the fact that patients were recruited from a tertiary referral center with many insomnia patients showing high symptom load and a severe degree of insomnia. This is illustrated by the finding that the sensitivity and specificity of the PSQI in our samples were similarly high and even higher than in the original study [35]. Final conclusions about the sensitivity and specificity of the RIS would therefore require additional studies in independent samples. However, it should be noted that the RIS has neither been designed nor validated as a tool for the diagnosis of PI or for differentiating between subtypes of insomnia.

Component analysis revealed that the RIS differentially assesses four factors. Besides “sleep depth” and quantitative aspects of sleep, the factor “fearfully focussing on insomnia” is especially interesting for an insomnia scale. We consider that the combination of psychological aspects that characterize psychophysiological insomnia as well as qualitative and quantitative aspects of sleep all represented in one short scale is the innovative aspect of this scale when compared to existing insomnia scales. Although this multifaceted structure of the RIS indicates its potential for differential assessment of different dimensions of insomnia (sleep quality, sleep quantity, focus on insomnia and medication intake) it should be noted that the RIS has been developed and validated as a single scale and should also be primarily used so.

The RIS was sensitive for detecting improvements after CBT-I in sleep parameters and target symptoms. Both the PSQI and RIS were comparable in measuring effects of CBT-I on sleep latency, sleep duration and hypnotic intake. In addition, the RIS detected effects in target symptoms of CBT-I, especially in thinking about sleep. This is an important result, since modification of dysfunctional beliefs is one of the central aspects of CBT-I [37]. Effects were also seen in sleep-related fear, indicating the sensitivity of the RIS to the emotional aspects of PI. The changes observed in the perception of sleeplessness suggest that the RIS also covers sleep misperception, which is regarded as one of the central aspects of PI [14,38] . Thus similar to the ISI [6] and the Dysfunctional Beliefs and Attitudes about Sleep Scale [27], the RIS is sensitive to psychotherapy-specific target symptoms and is therefore proposed as a complementary scale with a special focus on psychological symptoms of PI.

Beyond measuring severity of PI, RIS (and especially its different dimensions) may be a useful instrument in disentangling the psychological symptoms from the symptoms of the organic sleep disorders. In a recently published study, we showed that psychophysiological insomnia-specific symptoms contribute to a less compliant attitude towards a treatment of sleep apnea with continuous positive airway pressure [39]. This study was done with the RIS and showed new aspects in comparison to another study that was performed with the ISI earlier [40]. While Nuygen et al., could not see any impact of insomnia symptoms on CPAP compliance with the ISI, two items in the RIS (“I feel that I have not slept all night” and “I am afraid to got to bed because of my disturbed sleep”) specifically correlated with compliance [39].

There are limiting factors in this study design. In this study the RIS has been validated using a sample of insomnia patients with a broad spectrum of severity, ranging from outpatients to patients who received inpatient CBT-I. This is reflected by the broad distribution of the scores (Figure 1). Nevertheless, a potential selection bias cannot be excluded since the sample stems from a specialized tertiary referral centre. Therefore evaluation of the RIS in other samples is strongly encouraged. For the English version normative studies are necessary. Further studies should also investigate the relation to objective sleep data and the comparability to other short insomnia scales recently published. Also the sensitivity of the RIS for other therapeutic interventions such as pharmacotherapy should be addressed. In addition it was not validated against objective measures such as actigrapy or polysomnography. However, since this scale focuses on psychological symptoms and since a wide range in objective sleep parameters in insomnia patients are known [41], we consider this rather a minor problem. Another problem is that this scale was not cross validated against a current insomnia scale. We chose the PSQI because in the evaluation period of the RIS there have been a lot more studies done on insomnia patients with the PSQI rather than the ISI. Furthermore, it was not our purpose to design an alternative to ISI, but rather a new measurement for psychophysiological symptoms and sleep parameters in a short scale with good feasibility.

## Conclusion

The RIS is a short, economic and valid instrument for measuring psychological and physiological aspects of PI. It specifically detects changes in target symptoms of CBT-I typical for PI and thus represents an especially well-suited instrument for assessing treatment effects of insomnia specific CBT in both research and clinical use.

## Abbreviations

CBT-I: Insomnia specific cognitive behaviour therapy; ICSD-2: International Classification of Sleep Disorders, Second Edition; ISI: Insomnia Severity Index; NORM: Sample of patients with PI in which normative data were obtained; PI: Psychophysiological insomnia; PSQI: Pittsburgh Sleep Quality Index; RIS: Regensburg Insomnia Scale; THERAPY: Sample of patients with PI in which sensitivity to therapy changes have been obtained.

## Competing interests

None of the authors has a conflict of interest with respect to this study.

## Authors’ contribution

TC, PG and GH designed the scale and the study, TC, CP and PG recruited and examined the patients and healthy controls, TC, PG, BL , RP and HL analyzed and interpreted the data. The manuscript was drafted by TC, PG, BL and GH, and all authors approved the final version of the manuscript.
